# Work-related complaints of arm, neck and shoulder among computer office workers in an Asian country: prevalence and validation of a risk-factor questionnaire

**DOI:** 10.1186/1471-2474-12-68

**Published:** 2011-04-04

**Authors:** Priyanga Ranasinghe, Yashasvi S Perera, Dilusha A Lamabadusuriya, Supun Kulatunga, Naveen Jayawardana, Senaka Rajapakse, Prasad Katulanda

**Affiliations:** 1 Diabetes Research Unit, Department of Clinical Medicine, Faculty of Medicine, University of Colombo, Sri Lanka; 2 Department of Clinical Medicine, Faculty of Medicine, University of Colombo, Sri Lanka

## Abstract

**Background:**

Complaints of arm, neck and/or shoulders (CANS) affects millions of computer office workers. However its prevalence and associated risk factors in developing countries are yet to be investigated, due to non availability of validated assessment tools for these countries. We evaluated the 1-year prevalence of CANS among computer office workers in Sri Lanka and tested the psychometric properties of a translated risk factor questionnaire.

**Methods:**

Computer office workers at a telecommunication company in Sri Lankan received the Sinhalese version of the validated Maastricht Upper Extremity Questionnaire (MUEQ). The 94 items in the questionnaire covers demographic characteristics, CANS and evaluates potential risk factors for CANS in six domains. Forward and backward translation of the MUEQ was done by two independent bi-lingual translators. One-year prevalence of CANS and psychometric properties of the Sinhalese questionnaire were investigated.

**Results:**

Response rate was 97.7% (n = 440). Males were 42.7%. Mean age was 38.2 ± 9.5 years. One-year prevalence of CANS was 63.6% (mild-53.7% and severe-10%). The highest incidences were for neck (36.1%) and shoulder (34.3%) complaints. Two factors for each domain in the scale were identified by exploratory factor analysis (i.e. work-area, computer-position, incorrect body posture, bad-habits, skills and abilities, decision-making, time-management, work-overload, work-breaks, variation in work, work-environment and social-support). Calculation of internal consistency (Cronbach's alpha 0.43-0.82) and cross-validation provided evidence of reliability and lack of redundancy of items.

**Conclusion:**

One year prevalence of CANS in the study population corresponds strongly with prevalence in developed countries. Translated version of the MUEQ has satisfactory psychometric properties for it to be used to assess work-related risk factors for development of CANS among Sri Lankan computer office workers.

## Background

Complaints of the arm, neck and/or shoulder (CANS) is defined as "musculoskeletal complaints of arm, neck and/or shoulder not caused by acute trauma or by any systemic disease" [1]. CANS affect millions of computer office workers in developed countries [2], and is the leading cause of occupational illness in the United States with related absenteeism and medical expenses costing the industry $45 to $54 billion annually [3]. There is an increasing use of computer systems in developing countries.

Sri Lanka, developing nation in South Asia having a population of about 19 million people with Sinhalese being the mother tongue of 82% [4]. Ten percent of households in Sri Lanka posses a computer, availability of computers in households is highest in the Western province (19 percent) [5]. The rapid economic development of recent decades has led to computer systems being increasingly utilized in state and private sector organizations to improve productivity. There are no published data on the extent of the work-related CANS in Sri Lanka and the South-Asian region.

Initially, most research on work-related neck and upper limb symptoms focused only on physical exposure [6]. However recent studies have demonstrated that CANS have a multi-factorial origin; possible risk factors are of a physical, psychosocial or personal origin [3]. The identification and measurement of the various risk factors for these complaints is an important initial step in recognizing high risk subgroups also for developing targeted and effective intervention plans. Thus, a validated study instrument that is able to assess both prevalence of CANS and evaluate risk factors would be valuable in countries like Sri Lanka where data on CANS is minimal. The objectives of the present study were to a) translate and validate the Musculoskeletal Upper Extremity Questionnaire (MUEQ - is used to assess the occurrence and nature of CANS and work-related physical and psychological risk factors for the development of CANS) and b) to assess the 1-year prevalence of CANS in the study population. The psychometric properties of the original version of the MUEQ questionnaire has already been reported [7]. The psychometric properties of the Sinhalese translation and prevalence of CANS in the study population are reported in the present paper.

## Methods

### Study population

The present study was conducted between January and February 2009. The study population consisted of 450 office workers who were invited to participate in the study from a telecommunication company in Colombo, the commercial capital of the country. Informed written consent was obtained from each study participant. To be included, an office worker had to be employed in the current position for at least twelve months and use computers to complete their job tasks for at least two hours per day. Participants were excluded based on the following criteria: (1) suffering from diseases affecting the muscluskeletal system such as Rheumathoid Arthritis, Osteoarthritis and other Connective Tissue Disorders; (2) having a previous surgery of the upper musculoskeletal extremity. Ethical approval for the study was obtained from the Ethics Review Committee of the Faculty of Medicine, University of Colombo, Sri Lanka.

### Study instrument

Items included in the questionnaire were derived from the MUEQ which was developed in 1999. The psychometric properties of the original questionnaire have been investigated and were found to be valid and reliable [6]. The MUEQ was translated into Sinhalese with a forward and backward translation procedure. Two independent professional bilingual translators (English-Sinhala) translated the original scale once. They were encouraged to strive for idiomatic rather than word-for-word translation. This Sinhalese version was then reviewed by a team of experts comprising of one sworn translator, two physicians and one rheumatologist to assess the necessity of performing a cultural adaptation and to fine-tune it for use among Sri Lankan office workers. A backward translation to English of this reviewed Sinhalese version was done and compared with the original English version to verify that the meaning of each item of the scale was preserved. This comparison was performed by the initial team of experts.

The Sinhalese version consists of six pages with 94 items with completion time of approximately 30 minutes. The Sinhalese questionnaire covers demographical information of the subjects under study in addition to the six main domains of risk factors as in the MUEQ. These were the following domains: (1) work station; (2) body posture; (3) job control; (4) job demands, (5) break time; and (6) social support. The frequency and nature of upper extremity complaints and quality of the work environment was also assessed. Further items specified the clinical manifestations of the complaint (i.e. tingling, numbness, weakness, swelling, stiffness, fatigue, continuous pain and change in skin colour or temperature). All items were rephrased as statements in either a five point scale (always-never) or a dichotomous statement (yes-no). All data were double entered and cross checked for consistency. Data were analyzed using SPSS version 14 (SPSS Inc., Chicago, IL, USA) statistical software package. A p-value ≤ 0.05 was considered statistically significant.

### Calculation of the prevalence

The prevalence of CANS over the past twelve months lasting for at least one week were computed including 95% confidence intervals for each upper musculoskeletal body region (neck, shoulder, arm, elbow, hand and wrist). The following complaints of upper musculoskeletal extremity were considered in the MUEQ: pain, fatigue and exhaustion, stiffness, numbness, tingling sensation, weakness and swelling. Participants who reported complaints in the upper extremity were classified into two sub-groups: (1) mild cases: subjects who reported pain or/and complaints in one or more of the body regions neck, shoulder, hand, wrist and elbows for at least seven days during the preceding 12 months; (2) severe cases: subjects who reported pain or/and complaints in one or more of the body regions neck, shoulder, hand, wrist and elbows for at least seven days during the preceding 12 months while the pain was chronic (lasting for over a month) and present even after a short rest. The prevalence of complaints for mild and severe cases for the past twelve months was computed for males and females including 95% CI.

To investigate the extent of the spread of symptoms over the upper extremity prevalence including 95% CI were calculated for the following combinations of body regions: (1) Neck, shoulder, upper arm, elbow, lower arm, hand and wrist symptoms; (2) Neck, shoulder and upper arm symptoms, (3) Neck and shoulder symptoms, (4) Lower arm, hands and wrist symptoms and (5) Hands and wrist symptoms.

### Validation of the questionnaire

Exploratory factor analysis is a technique used to analyze interrelations among a large number of items while trying to explain these items in terms of their common underlying dimensions [8]. We conducted Principal Component Analysis (PCA) with Varimax rotation to divide the items for each of the six domains into two factors. The number of factors retained was derived by considering the magnitude of the eigenvalues, Kaiser's (1960) eigenvalues (greater than 1) rule, the proportion of variance extracted, item content, and the interpretability of the resulting factors. As for factor loading after the Varimax rotation, items with a factor loading less then 0.5 on all factors were excluded. Further, each factor had to comprise at least three items. If the results indicated more than two factors, a forced two factor analysis was performed.

We investigated the internal consistency by calculating Cronbach's alpha and by calculating item-total correlations for each factor that was identified with the factor analysis, an Alpha greater than 0.70 was considered acceptable and optimal item-total correlation was considered to be between 0.2 and 0.5 [8].

In order to test the stability of the factor structure cross-validation was carried out. Cross-validation, is the statistical method of partitioning a sample of data into subsets such that the analysis is initially performed on a single subset, while the other subset(s) are retained for subsequent use in confirming and validating the initial analysis [9]. For this purpose a sub-sample (n = 220) was randomly selected from the study population.

## Results

### Demographic characteristics

Four hundred and forty computer office workers out of the 450 invited for the study responded to the questionnaire (response rate of 97.7%). Mean age was 38.2 (SD ± 9.5) and 188 (42.7%) were males; and 29.3% were aged between 40 and 49 years, 31.4% of the males and 27.4% of the females belonged to this age group. Thirty percent of the study population had worked between 1 to 5 years in their current position. Of the female participants, 66.3% worked 6 to 9 hours per day with a computer compared to 43.1% of the male participants and 56.4% of the entire study population (Table 1).

**Table 1:** Characteristics of the study population

	All	Males	Females
Age			
20 - 29 years	104 (23.6%)	41 (21.8%)	63 (25.0%)
30 - 39 years	116 (26.4%)	44 (23.4%)	72 (28.6%)
40 - 49 years	129 (29.3%)	59 (31.4%)	69 (27.4%)
≥50 years	91 (20.7%)	44 (23.4%)	48 (19.0%)

**Number of working years in current position**			
1 to 5 years	132 (30.0%)	63 (33.5%)	69 (27.4%)
6 to 10 years	107 (24.3%)	47 (25.0%)	60 (23.8%)
11 to 15 years	94 (21.4%)	40 (21.3%)	54 (21.4%)
15 years and more	107 (24.3%)	38 (20.2%)	69 (27.4%)

**Number of working hours with computer/day**			
2 to 5 hrs	107 (24.3%)	63 (33.5%)	44 (17.5%)
6 to 9 hrs	248 (56.4%)	81 (43.1%)	167 (66.3%)
> 9 hrs	85 (19.3%)	44 (23.4%)	41 (16.3%)

### Prevalence of CANS

The 1-year prevalence of CANS in the study population was 63.6%. Prevalence of mild cases was 53.7% (males Vs. females; 55% Vs 52% respectively). The 1-year prevalence of severe cases was 10% (males 11.7%, females 8.8%). The most commonly reported complaints were neck and shoulder symptoms (37.1% and 34.3% respectively), followed by hand, wrist and upper arm complaints (23.6%, 21.4% and 18.6% respectively) and elbow complaints and lower arm (11% and 9% respectively) (Table 2).

**Table 2:** One year prevalence of CANS lasting for at least one week during the previous year

Complaint	Number of subjects with complaints	All Prevalence (95% CI) (n = 440)	Males Prevalence (95% CI) (n = 188)	Females Prevalence (95% CI)(n = 252)
Neck complaints	163	0.37 (0.29 to 0.45)	0.38 (0.26 to 0.51)	0.36 (0.26 to 0.47)
Shoulder complaints	150	0.34 (0.26 to 0.42)	0.33 (0.22 to 0.46)	0.35 (0.25 to 0.46)
Upper arm complaints	84	0.19 (0.12 to 0.25)	0.18 (0.10 to 0.29)	0.19 (0.11 to 0.28)
Elbow complaints	48	0.11 (0.06 to 0.16)	0.07 (0.02 to 0.14)	0.14 (0.07 to 0.22)
Lower arm complaints	40	0.09 (0.05 to 0.15)	0.08 (0.03 to 0.16)	0.10 (0.04 to 0.17)
Wrist complaints	92	0.21 (0.15 to 0.28)	0.12 (0.05 to 0.21)	0.29 (0.19 to 0.39)
Hand complaints	106	0.24 (0.17 to 0.31)	0.18 (0.10 to 0.29)	0.28 (0.18 to 0.38)
Mild cases	238	0.54 (0.45 to 0.62)	0.55 (0.42 to 0.67)	0.52 (0.41 to 0.63)
Severe cases	44	0.10 (0.06 to 0.15)	0.12 (0.05 to 0.21)	0.09 (0.04 to 0.16)

The 1-year prevalence of complaints of the various upper extremity body regions (except for neck complaints) was greater for females than for males (Table 2). This difference was statistically significant for the wrist complaints (p < 0.05). In both males and females complaints of the "right side" were reported more frequently than for the "left side" (Table 3). Majority of the study population were right-handed (90%/n = 396).

**Table 3:** Number and percentage of CANS during the previous year, enduring one week distributed by anatomical location

	Complaints of each anatomical area (%)					
	
	Shoulder	Upper Arm	Elbow	Lower arm	Wrist	Hand
**Males (N = 188)**						
Right side	25 (13.3)	19 (10.1)	32 (1.7)	94 (5.0)	32 (1.7)	32 (1.7)
Left side	6 (3.2)	94 (5.0)	32 (1.7)	32 (1.7)	32 (1.7)	94 (5.0)
Both sides	28 (14.9)	6 (3.2)	6 (3.2)	32 (1.7)	16 (8.5)	16 (8.5)

**Female (N = 252)**						
Right side	28 (11.1)	26 (10.3)	16 (6.3)	13 (5.2)	38 (15.1)	38 (15.1)
Left side	3 (1.2)	9 (3.6)	6 (2.4)	3 (1.2)	9 (3.6)	6 (2.4)
Both sides	53 (21.0)	6 (2.4)	9 (3.6)	6 (2.4)	13 (5.2)	22 (8.7)

Complaints of the entire upper extremity were reported by 2.8% of the study population, whereas 8.6% reported complaints of the neck, shoulder and upper arm and 22.1% complained of neck and shoulder complaints.

### Psychometric properties of the questionnaire

Results from the factor analysis indicated that each domain included two factors accounting for approximately 40% of the variance. Cronbach's alpha coefficients for the majority of the factors in the questionnaire were greater than the accepted number of ≥ 0.70. However, some of the factors (i.e. computer position, work area, variation in work and decision making) showed an lower alpha and showed suboptimal item-total correlation (below 0.2).

### Results of the cross-validation

We found that the number of factors, the factor structure and factors loadings were for the greater part comparable between the first randomly created sub-sample (n = 220) and the total sample (n = 440). Differences were found in the 'social support' domain. The items " I alternate in my job task", "I perform job tasks without a computer" and "after two hours work I take a break for at least 10 minutes" loaded positively on the first factor in the randomly selected sub-sample; however, the same items loaded highly on the second factor in the total sample analysis. No further differences were found between the results of the total sample analysis and the randomly selected sub-sample. We therefore present the results of the factor analyses as applied to the total sample (Table 4). The results of the internal consistency analyses and item-total correlations are presented in table 5.

**Table 4:** Factor loadings and orthogonal VARIMAX rotation

Domain	Abbreviated item description	Factor1	Factor2
**Work Station**		**Work area**	**Computer position**

My desk at work has a suitable height		0.73	0.27
I have enough space to work at my office		0.63	0.27
I can adjust my chair height		0.70	-0.32
My keyboard is placed directly in front of me		-0.05	0.83
The screen is placed directly in front of me		0.14	0.67
When I use the mouse device my arm is supported by the Table		0.17	0.53
Eigenvalue		1.45	1.54
% of Variance		22.0	20.7

**Body Posture**		**Incorrect body posture**	**Bad habits**

When I work my head is bent		0.60	-0.01
When I work my head is twisted towards the left or right		0.72	0.15
When I work my body is twisted towards the left or right		0.74	0.13
My Trunk is in asymmetrical position		0.78	0.24
During my work I sit for long hours in one position		-0.01	0.77
For more than 2 hours/day I work with lifted shoulders		0.02	0.64
During my work I sit in an awkward posture		0.55	0.57
In work I perform repetitive tasks		0.12	0.73
I find my job physically exhausting		0.20	0.80
Eigenvalue		2.90	2.63
% of Variance		26.4%	23.9%

**Job Control**		**Skills and abilities**	**Decision making**

My work develops my abilities		0.79	0.29
In my work I have the chance to learn new things		0.83	0.26
I have to be creative in my work		0.82	0.02
I undertake different tasks in my work		0.74	-0.02
I decide how to perform my job task		0.04	0.72
I participate with others in decision taking		-0.03	0.51
I decide my own task changes		0.10	0.64
I determine the time & speed job tasks		0.22	0.72
Eigenvalue		2.65	2.06
% of Variance		29.4	22.8%

**Job Demands**		**Time management**	**Work overload**

I work under extensive work pressure		0.79	-0.09
I find it difficult to finish my job tasks on time		0.70	-0.38
I take extra hours to finish my job tasks		0.78	-0.24
I have don't have enough time to finish my job task		0.83	-0.13
At work I speed to finish my tasks on time		0.36	0.88
I find my work tasks difficult		0.14	0.73
I have too many job tasks		0.40	0.62
Eigenvalue		3.55	1.18
% of Variance		33.7	26.1

**Break Time**		**Work breaks**	**Variation in work**
		
I can plan my work breaks		0.84	0.14
I can decide when to take a break		0.82	0.20
I can divide my work time		0.81	0.06
I alternate in my job task		0.18	0.74
I perform job tasks without a computer		-0.18	0.78
After two hours work I take a break for at least 10 minutes		0.23	0.58
Eigenvalue		2.48	2.01
% of Variance		30.9%	25.1%

**Social Support**		**Work environment**	**Social Support**

The work flow goes smoothly		0.81	0.10
I can ask and enquire about my work		0.78	0.24
My work atmosphere is comfortable		0.56	0.32
If I made a mistake in my work task I find support from my colleagues		0.10	0.81
If I made a mistake in my work task I find support from my supervisors		0.13	0.81
My colleagues are friendly		0.46	0.52
Eigenvalue		2.14	2.11
% of Variance		26.8%	26.3%

**Table 5:** Internal consistency and Item-total correlation of the twelve Factors

Domain	Factors	Internal consistency (Cronbach's alpha)	Item-total correlation (Min-Max)	Items numbers
**Work Station**	Factor 1: Work area	0.46	0.18 to 0.39	9,10,15
	Factor 2: Computer position	0.43	0.14 to 0.37	11,13,14

**Body Posture**	Factor 3: Incorrect body posture	0.74	0.27 to 0.68	23,24,25,26
	Factor 4: Bad habits	0.76	0.45 to 0.66	17,18,19,20,21

**Job Control**	Factor 5: Skills and abilities	0.82	0.53 to 0.74	32,33,34,35
	Factor 6: Decision making	0.60	0.26 to 0.49	27,28,29,30

**Job Demands**	Factor 7: Time management	0.82	0.61 to 0.72	36,37,38,39
	Factor 8: Work overload	0.65	0.30 to 0.57	40.41.42

**Break Time**	Factor 9: Work breaks	0.80	0.61 to 0.70	43,44,45
	Factor 10: Variation in work	0.60	0.29 to 0.46	47,48,49

**Social Support**	Factor 11: Work environment	0.73	0.47 to 0.63	51, 52, 54
	Factor 12: Social support	0.68	0.34 to 0.63	55,56,57

#### Work station

The first domain consisted of seven items and assessed the work station (i.e. table, chair and computer placement). Two factors were extracted. Examination of the factor loadings showed that the item "The chair I use during work supports my lower back" loads poorly on both factors, therefore it was excluded. The first factor held three items ("My desk (table) at work has a suitable height", "I have enough space to work at my office" and "I can adjust my chair height"). This first factor, which was related to work area, accounted for 22.0% of the total variance and had a Cronbach's alpha of 0.46 while values of item-total correlations varied between 0.18 and 0.39. The second factor included three items ("My keyboard is placed directly in front of me", "The screen is placed directly in front of me" and "When I use the mouse device my arm is supported by the table"). They were related to the computer position and accounted for 20.7% of the total variance. This factor had a Cronbach's alpha of 0.43 and the item-total correlation was between 0.14 and 0.37.

#### Body Posture

The second domain evaluated body posture and consisted of 11 items. Two factors were extracted. The Scree plot and the examination of the rotated factor loadings showed that two items ("During my work I keep a good work posture" and "When I key my hand is placed in a straight line with my lower arm") load poorly on both factors justifying deletion of these items. The first factor, included four items related to incorrect body posture ("When I work my head is bent", "Head is twisted towards the left or right", "Trunk is twisted towards the left or right" and "My trunk is in an asymmetrical position") accounting for 26.4% of the total variance, with a Cronbach's alpha of 0.74, and item-total correlations ranging from 0.27 to 0.68.

The second factor included five items related bad habits ("During my work I sit for long hours in one position", "For more than two hours per day I work with lifted shoulders", "During my work I sit in an awkward posture", " In work I perform repetitive tasks" and "I find my job physically exhausting") accounting for 23.9% of the total variance. Cronbach's alpha for this factor was 0.76 and the item-total correlations ranged from 0.45 to 0.66.

#### Job Control

The job control domain included 9 items. Two factors were identified in this domain. The Scree plot and the examination of the rotated factor loadings showed that the item "I solve work problems by my self" loaded poorly on both factors justifying its' deletion. The rotated factor loadings indicated that the first factor on skills and abilities contained four items ("My work develops my abilities", "In my work I learn new things", "I have to be creative in my work" and "I undertake different tasks in my work") accounting for 29.4% of the total variance with a Cronbach's alpha of 0.82 and item-total correlations ranging from 0.53 to 0.74. The second factor on decision making contained four items ("I decide how to perform my job task", "I participate with others in decision taking", "I decide my own task changes" and "I determine the time and speed of job tasks"). This accounted for 22.8% of the total variance. Cronbach's alpha was 0.60 and the item-total correlations ranged from 0.26 to 0.49.

#### Job Demands

The domain job demands consisted of 7 items. The Scree plot results identified two factors. The first factor (i.e. time management) included four items ("I work under extensive work pressure", "I find it difficult to finish my job tasks on time", "I take extra hours to finish my job tasks" and "I have don't have enough time to finish my job task "). This accounted for 33.7% of the total variance, Cronbach's alpha was 0.82 and the item-total correlations ranged from 0.61 to 0.72. The second factor (i.e. work overload) held three items ("At work I speed to finish my tasks on time", "I find my work tasks difficult" and "I have too many job tasks"). This accounted for 26.1% of the total variance and Cronbach's alpha was 0.65. Item-total correlations ranged from 0.30 to 0.57.

#### Break Time

Break time during working hours was investigated by 8 items. The Scree plot results identified two factors. The Scree plot and the examination of the rotated factor loadings showed that the two items ("I alternate in my body posture." and "I find my work breaks sufficient") loaded poorly on both factors justifying their deletion. The first factor on work breaks holds three items ("I can plan my work breaks.", "I can decide when to take a break" and " I can divide my work time ") which accounts for 30.9% of the total variance. Cronbach's alpha of the autonomy factor was 0.80 and the item-total correlations ranged from 0.61 to 0.70. Three items related to variation in work load highly on the second factor ("I alternate in my job task", "I perform job tasks without a computer" and "after two hours work I take a break for at least 10 minutes") accounting for 25.1% of the total variance, with a Cronbach's alpha of 0.60 and item-total correlations ranging from 0.29 to 0.46.

#### Social Support

Eight items investigated the relationship among co-workers and between workers and supervisors. The Scree plot indicated that two factors (i.e work environment and social support) were to be retained. Two items ("My work tasks depends on other colleagues." and "My supervisors are friendly") loaded poorly on both factors and thus was excluded. The rotated factor loadings indicated that three items load highly on the first factor on work environment ("The work flow goes smoothly", "I can ask and enquire about my work" and "My work atmosphere is comfortable") accounting for 26.8% of the total variance. Cronbach's alpha was 0.73 and item-total correlations ranged from 0.47 to 0.63. The other three items ("If I made a mistake in my work task I find support from my colleagues", "If I made a mistake in my work task I find support from my supervisors" and "My colleagues are friendly") were classified as being related to social support and accounted for 26.3% of the total variance. Cronbach's alpha was 0.68 and item-total correlations ranged from 0.34 to 0.63.

## Discussion

This is the first study investigating the prevalence of CANS in a population of computer office workers in Sri Lanka. The 1-year prevalence of neck and shoulder complaints in the study population was higher than the prevalence of arm, hand and elbow complaints. A statistically significantly higher proportion of the study population (63.6%) reported CANS of at least one week duration over a one-year period. These figures are comparable to results of previous studies conducted in Netharlands and Germany [7,10]. However, the 1-year prevalence of CANS in an open population of adults (36.8%) was much lower than among the computer office workers in the present and other similar studies [11]. This further highlights the importance of work-related repetitive injury and/or environmental risk factors in the genesis of CANS among computer office workers. The majority of the participants in our study were classified as mild cases, while only 44 cases (10%) were classified as severe cases. In a study, conducted in Denmark, Andersen and colleagues found that only small proportions (<3%) of participants reported moderate to severe acute and chronic neck and shoulder pain [12].

We have attempted to accurately examine the measurement properties of the Sinhalese version of the MUEQ. The translation and adaptation of pre-existing questionnaires have two advantages: translated questionnaires provide an efficient way to have a valid and reliable domain that needs to be measured in the targeted language; if the translation shows good psychometric properties, such translated instruments can be used in international comparative studies. In addition using a validated translation of a single questionnaire in research on upper-extremity disorders makes it easier to compare the results between different populations. However, the assumption is that simple translation is usually successful if the culture of the target population is similar to that of the original population. Because the Sri Lankan and the Dutch cultures are different, we strived for an idiomatic rather than word-for-word translation during the translation process. The results of the psychometric analyses indicated that the two scales were psychometrically similar [7]. In both questionnaires twelve factors were extracted, explaining approximately 50% of the variance in the original version of the MUEQ compared to 40-50% in the Sinhalese version of the MUEQ. Cronbach's alpha coefficients in the original version of the MUEQ ranged from 0.54 to 0.85 compared to 0.43 to 0.82 in the Sinhalese version. In general, cultural differences did not hinder the use of the translated version among the Sinhalese cohort. Thus, one can postulate that physical and psychosocial factors related to computer office work are not perceived differently by different cultures. Whether the scales identified by the factor analyses in this study are indeed risk factors for the development of CANS in computer workers, will be the topic of research of a prospective study conducted by our group.

The disability of arm, shoulder and hand (DASH) questionnaire, is another validated instrument developed for the assessment upper musculoskeletal symptoms [13,14]. Initially this instrument was developed to measure disability in patients with arm, shoulder and hand disorders. However, patients with shoulder, arm, or hand complaints frequently report neck complaints as well. Therefore, a valid and responsive questionnaire designed for the whole upper extremity including the neck, would be very useful and practical in upper-extremity research [13]. The DASH questionnaire has shown to contribute to this statement. It is a valid questionnaire to measure disability in patients with upper-extremity disorders including the neck [13,14]. Although the DASH demonstrated concurrent validity in patients with neck pain [14], more studies are needed to assess the reliability, validity, and responsiveness of the DASH to measure disability in patients with isolated neck complaints [13]. The MUEQ is another recently developed instrument that could be used for the assessment of the whole upper musculoskeletal extremity. However, its reliability and validity to measure complaint of the different regions of the upper musculoskeletal extremity including isolated neck complaints requires further study.

The 1-year prevalence of CANS among computer office workers in the present study and causal relationships reported in the literature are often derived from cross-sectional studies. However, in order to accurately investigate causal relations between both physical and psychosocial risk factors and CANS prospective cohort studies are needed. An example of such a cohort study is the NUDATA study among Danish computer workers, which showed that mouse and keyboard use were associated with an increased risk of carpal tunnel syndrome, elbow and wrist/hand symptoms, forearm pain, and neck and shoulder symptoms [15-19].

Data on the prevalence of musculoskeletal disorders have been collected for several decades in developed countries. Studies on the epidemiology of CANS are mostly restricted to high-income countries, comprising less than 15% of the world population [20]. The current study documents that the prevalence of CANS in computer office workers in Sri Lanka seems to correspond with prevalence of CANS found in developed countries. Furthermore, the study presents a valid and reliable Sinhalese questionnaire to be used to assess work-related risk factors for the development of CANS. Nevertheless, the psychometric properties of this questionnaire were studied in employees without severe musculoskeletal complaints. Further evaluation of the psychometric properties of the questionnaire via studies in other populations may therefore be useful. In addition although physical risk factors may be similar in many populations worldwide, associated psychosocial risk factors will differ from one populations to another, thus in this context the South-Asian population could be different from counterparts in rest of the world. Indeed studies from the region have demonstrated that they demonstrate a collective pattern, rather than an individualistic pattern, of social interaction [21].

The present study has several limitations, the cross-sectional nature of the present study prevented accurate investigation of causal relations between both physical and psychosocial risk factors and CANS. Thus, in order to establish a causal relationship further prospective studies, controlling for confounder variables are required. In addition the study population was selected as a convenience sample as the primary purpose of the present study was questionnaire translation and validation. Thus, caution is required when generalizing the prevalence of work related CANS in the present study to other computer office workers.

## Conclusion

The estimated one year prevalence of CANS in computer office workers in Sri Lanka seems to correspond with the prevalence of CANS found in developed countries. We highlights the scientific methodology of translating and validating a questionnaire for the purpose of usage in countries where native language differs from the questionnaire's original language. In addition we present a valid and reliable Sinhalese questionnaire to be used to assess work-related risk factors for the development of CANS among Sri Lankan computer office workers.

## Competing interests

The authors declare that they have no competing interests.

## Authors' contributions

SR, DAL and NJ made substantial contribution to conception and study design. SK and NJ were involved in data collection. YSP and PR were involved in refining the study design, statistical analysis and drafting the manuscript. PK, PR and YSP critically revised the manuscript. All authors read and approved the final manuscript

## Pre-publication history

The pre-publication history for this paper can be accessed here:

http://www.biomedcentral.com/1471-2474/12/68/prepub
